# Serial Coincubation Enrichment Technique (SCET): Rapid isolation of bacterial biocontrol agents

**DOI:** 10.1016/j.mex.2026.103849

**Published:** 2026-02-26

**Authors:** Hemant Singh Maheshwari, Aakash Gour, Sanjeev Kumar, Laxman Singh Rajput, Jeberlin Prabina Bright, Rakesh Kumar Singh, Mahaveer Prasad Sharma, Kunwar Harendra Singh

**Affiliations:** a ICAR-National Soybean Research Institute, Indore, India; b University of Groningen, Groningen, the Netherlands; c ICAR-Central Arid Zone Research Institute, Jodhpur, India; d Tamil Nadu Agricultural University, Coimbatore, India; e Rajmata Vijayaraje Scindia Krishi Vishwavidyalaya, Gwalior, India

**Keywords:** Serial Coincubation Enrichment Technique (SCET), Rhizomicrobiome, Fungal phytopathogen, Bacterial biocontrol agent, Antagonistic, Dual assay

## Abstract

The Serial Coincubation Enrichment Technique (SCET) mimics the rhizomicrobiome interaction between antagonistic bacteria and the target fungal pathogen, and subsequent enrichment allows only the predominant bacteria with antagonistic activity to survive within 20 days or less. Here, soybean phytopathogens, *Rhizoctonia Solani, Colletotrichum truncatum*, and *Sclerotium rolfsii*, were taken separately. Their discs were placed in a 1:1 ratio of 100 ml nutrient broth and potato dextrose broth, grown for at least 24 h, and then incubated with a 1 g rhizospheric soil sample from healthy, disease-free soybean plants. The enrichment was performed by transferring 1 ml of the previous suspension to freshly prepared media containing the previously grown 24 h target pathogen for an additional 5 days, and this process was repeated twice. The final suspension was serially diluted and spread on nutrient agar containing nystatin.

• Coincubation of the targeted pathogen with the rhizospheric sample allows survival of bacteria with biocontrol properties against the target fungal pathogen within 20 days.

• SCET setup mimics the rhizomicrobiome or classical dual assay, where already existing pathogens interact with the antagonistic bacteria of the rhizosphere.

• Samples can be screened by comparing sole fungal growth in the medium against the fungal growth in a final enriched co-incubated setup.

## Graphical abstract

**Figure:**
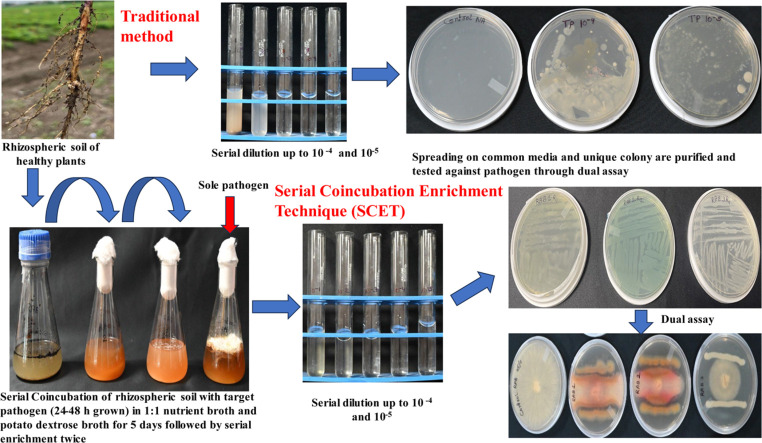

Isolation of bacterial antagonists against phytopathogens through Serial Coincubation Enrichment Technique (SCET)

**Specifications table**

**Table:** 

**Subject area**	Agricultural and Biological Sciences
**More specific subject area**	Plant pathology
**Name of your protocol**	Serial Coincubation Enrichment Technique (SCET)
**Reagents/tools**	Nutrient broth, Potato dextrose broth, Nystatin
**Experimental design**	Serial coincubation of rhizosphere soil samples with already grown targeted fungal phytopathogen allows antagonistic bacteria to survive against the pathogen. It mimics the rhizomicrobiome or classical dual assay test, which is carried out for screening bacteria with antagonistic properties. Samples can be screened based on significant differences in the weight of fungal biomass grown alone compared to fungal growth in finally enriched, coincubated samples.
**Trial registration**	Not applicable
**Ethics**	The present modified protocol did not involve human or animal subjects or data collected from social media platforms.
**Value of the Protocol**	Serial coincubation of rhizosphere samples with already grown targeted fungal pathogens enables the survival of bacteria with antagonistic properties. Screening of samples is possible by comparing the weight of the entire fungal biomass in coincubation against the sole fungal phytopathogen growth. Recovery of a few bacteria, which is easier in testing with antagonistic properties, compared to a large number of bacteria recovered through the traditional protocol.

## Background

Bacterial biocontrol agents have been isolated from the rhizosphere [1,2], endosphere [3,4], and phyllosphere [5] of healthy plants through conventional methods. The existing conventional methodology involves the isolation of bacterial biocontrol agents by serial dilution of rhizospheric soil or surface-sterilized crushed plant samples (endophytes) until 10^−5^ and 10^−6^ followed by spreading on common media like nutrient agar, Luria Bertini agar and tryptone soy agar followed by incubation at 28 ± 30 °C which led to the isolation of many bacteria, followed by further testing by dual culture assay, or the food poisoning technique to assess the bacteria with antagonistic properties against the target pathogens (Table 1) [3,4,6]. Furthermore, other biocontrol properties, such as siderophore production, HCN production, and the presence of cell wall-degrading enzymes (including cellulase, pectinase, chitinase, and protease), were also assessed [7].

**Table 1:** Differences between SCIT and traditional approaches.

Sr no	Serial Number	SCIT	Traditional approaches
**1**	**Methodology**	It mimics the rhizomicrobiome or classical dual assay, in which a target pathogen interacts with bacterial antagonists present in rhizospheric samples. Further enrichment eliminates non-antagonist bacteria.	Serial dilution of samples up to 10^−5^ or 10^−6^, followed by spreading in a common media
**2**	**Number of bacteria per sample**	Coincubation followed by serial enrichment yields only 1–2 bacterial types.	More than 10 types of bacteria appear on each Petri dish.
**3**	**Maintenance of culture**	Few cultures are easily maintained	A large number of bacteria, including non-antagonists, need to be maintained until screening.
**4**	**Recovery and type of bacteria**	Only the potential bacteria with antagonistic properties recovered	A large number of bacteria were recovered, which require further screening to identify the potential ones.
**5**	**Sample screening**	Possible by comparing the dry weight of the final coincubated samples against the sole target pathogen grown in the media	Not possible
**6**	**Rhizocompetence**	As recovered bacteria are rhizocompetent	Screening needed for rhizocompetent traits
**7**	**Resources**	Fewer resources, time, and labour are required for screening	Require greater resources, time, and labor-intensive
**8**	**Time**	20 days	2–4 months
**9**	**Limitation**	A few neutral or synergistic bacteria also grow, which need to be screened.	Many bacteria with non-antagonistic properties emerge, requiring more time and resources for screening.
**10**	**Future implication**	The final coincubated suspension can be used to isolate actinomycetes by placing it on actinomycete agar supplemented with an antibiotic. Similarly, other microorganisms, including fungal antagonists, may be recovered.	A separate protocol needs to be planned to get different types of antagonists.

In the past, targeted isolation of biocontrol agents was attempted by co-culturing the pathogen with the plant sample. They hypothesized that plants harbor symbiotic or associated beneficial bacteria that confer tolerance to infection by plant pathogens. However, in this case, many bacteria grew on the petri dishes, similar to traditional methods, necessitating further screening with a dual assay [8]. Enrichment of the sample in chitin as the sole source [9] or iron-free succinate media for siderophore-producing bacterial antagonists [10] has also been attempted. However, many mechanisms are simultaneously acting against the phytopathogen.

In the current modified protocol, we co-incubated the rhizospheric sample of healthy soybean plants in a medium containing the 24–48 h fully grown target fungal phytopathogen for five days with two consecutive transfers. We artificially mimic the rhizomicrobiome, enabling antagonistic bacteria from rhizosphere samples to interact with the target pathogen. It allows the growth and survival of bacteria with antagonistic properties or neutral bacteria, which can survive in media containing metabolites produced by the dominant fungal phytopathogen. This modified protocol was developed when plant growth-promoting bacteria were tested against the soybean phytopathogen *Rhizoctonia solani* in a dual assay. Then, bacteria with biocontrol properties against the target pathogen first occupy the entire Petri dish. The target fungal pathogen becomes blackish, likely due to melanin production in response to stress caused by the biocontrol action of the bacteria [11]. Therefore, we hypothesized that if both the target phytopathogen and the rhizospheric soils are co-incubated, then only those bacteria will survive that have the capacity to outcompete for space and nutrients, and to survive in the fungal exometabolites environment. Further, sample carrying antagonistic properties against the target phytopathogen would decrease the fungal biomass (Table 1).

Additionally, multitrophic interactions may occur, such as neutralism, competition, commensalism, and antagonism, resulting in the survival of only certain competent bacteria, while others die out [12]. Such surviving bacteria may be more effective at controlling the target phytopathogen due to their higher rhizocompetence. Here, the modified system is essentially a three-consecutive dual assay, which selectively favors bacteria with antagonistic properties and eliminates others.

### Description of protocol

In the modified protocols, we designed a medium containing a 1:1 ratio of nutrient broth (NB) and potato dextrose broth (PDB) to allow equal growth of both bacteria and fungi. Firstly, the medium was prepared, and then the target fungal phytopathogen disc was inoculated into the medium at 30 ± 2 °C for 24–48 h, depending upon the growth of the target fungal phytopathogen, so that the target fungus would dominate in the medium and prevent other soil fungi present in the sample from becoming dominant.

Then, 1 g of the rhizospheric soil sample from a healthy soybean plant was added to the medium and incubated for 5 days at 30 ± 2 °C. After 5 days, 1 ml of the suspension was transferred to fresh medium containing the target fungal pathogen, which was grown for 24–48 h. This step was performed at least twice, which selectively favored the growth and survival of antagonistic bacteria. The final flask suspension was then serially diluted up to 10^−5^, and 100 µl was spread onto a nutrient agar medium amended with antifungal nystatin, which inhibited fungal growth. The unique bacterial colony appearing on the petri dishes was purified by the streak plate method on nutrient agar. In this protocol, only 1–2 bacteria appeared on the petri dish per sample, which may be antagonistic to the target pathogen. We preferentially selected fast-growing bacteria that possess ideal characteristics to compete with the target phytopathogen (Table 1). Further, these recovered bacteria were tested against the target pathogen through a dual culture assay. This SCET protocol yields fewer bacteria with biocontrol properties compared to the conventional method. Screening these bacteria for their antagonistic properties against the target pathogen requires significantly less time, labor, and laboratory resources.

A recovered bacterium, obtained using a modified protocol, may exhibit greater activity in controlling the disease caused by the pathogen, as it has already interacted with it during isolation. The artificial rhizomicrobiome interaction setup conditions can be altered, such as pH and temperature, to suit the prevailing conditions of the disease in any region. For example, in the present case, a pH of 8.00 and a temperature of 28–30 °C were maintained to mimic these conditions, enabling simultaneous screening of the bacteria.

Another advantage of the protocol is the ability to screen samples at the coincubation stage, unlike the traditional protocol. Here, the entire media of the final coincubated samples were strained to discard the liquid medium, and the retained solid content was oven-dried at 65 °C. The solid dry weight of the coincubated sample was then measured and compared with the dry weight of the sole fungal pathogen grown alone in the medium. If the weight of the co-incubated sample is significantly lower than that of the sole fungal pathogen grown alone, the sample may carry potential antagonistic bacteria. In this step, we can screen a large number of samples and select those that significantly reduce fungal weight in the coincubation compared to the sole fungal pathogen, thereby obtaining efficient antagonistic bacteria.

### Equipment and consumables required

Autoclave

Laminar air flow

Rotary incubator shaker, pH meter

Hot air oven

Bacteriological filter 0.22 µm

Nystatin (Himedia)

Nutrient broth (Himedia)

Potato dextrose broth (Himedia)

Agar-agar (Himedia)

Erlenmeyer culture flask with vent cap, flat bottom (250 ml capacity)

Micropipette (100–1000 µl) Single channel

Autoclavable Micro Pipette tips (1 ml)

Analytical balance 220 g/0.1 mg capacity

Cork borer

Petri dish

Cell spreaders

Inoculating loops and needles

Whatman filter paper

Glass funnel

### Steps

1 Three different soybean phytopathogens, namely *Rhizoctonia solani* (PV536972),*Colletotrichum truncatum* (OR405112), and *Sclerotium rolfsii,* were obtained from ICAR-NSRI, Plant Pathology lab.
2 Nutrient broth (50 ml) and potato dextrose broth (50 ml) media were separately prepared and mixed, and then the media were autoclaved.
3 One disc of 10 mm size of pathogen (4 days old grown *Rhizoctonia solani* and *Sclerotium rolfsii,* and 9 days old of *Colletotrichum truncatum*) was inoculated into the three different flasks containing the media using a cork borer and allowed to grow for 24 - 48 h at 28–30 °C in a shaker with 120 rpm. Since some fungal phytopathogens are slow growers, like *Colletotrichum trucatum*, then 48 h is sufficient; however, in the case of fast growers like *Rhizoctonia solani* or *Sclerotium rolfsii*, 24 h is sufficient.
4 Then, rhizospheric samples of healthy & disease-free soybean plants were taken, and 1 g of soil was directly inoculated into all three media that already contained 24–48 h-grown fungal cultures.
5 Two successive transfers of 5 days each with 1 ml suspension were carried out into a fresh medium that already contained 24–48 h-grown target fungal phytopathogen growth for serial enrichment.
6 From the final serially enriched flask, 1 ml was taken and serially diluted to 10^−1^,10^−2^,10^−3^,10^−4^ and 10^−5^, and 100 µl from 10^−5^ was spread onto nutrient agar plates amended with antifungal antibiotic nystatin 50 µg ml^−1^, which selectively allows the growth of bacteria.
7 Remaining content in the final flask was strained through Whatman filter paper, and solid content was collected and placed in the hot air oven at 65 °C to get the dry weight for screening the samples.
8 Bacteria appearing on the Petri dish within 24 h were preferentially selected as predominant bacteria are of interest.
9 These bacteria were directly used for a dual culture assay against the target phytopathogens.
10 Further studies on properties like HCN production [13], Siderophore production and cell wall-degrading enzymes were employed to support the mechanisms of antagonism.

**Two criteria to be considered during the final observation:**

• **Dry weight of entire biomass:** The dry weight of the entire biomass after straining the media grown after 5 days of final coincubation in the flask was recorded. If the dry biomass of the final coincubated flask is significantly lower than that of the sole fungal growth culture, then the sample may contain potential bacteria with antagonistic activities. If the entire biomass is not significantly reduced, then the bacteria present in the medium lack a potential antagonist, and the samples can be rejected directly. Moreover, we can also visually screen them by comparing fungal growth in a coincubated flask with that of the sole fungal pathogen grown alone. Since the cottony growth of the fungus can be easily seen on the surface of the medium.
• **Dual culture assay:** Finally, the dual culture assay is done as a confirmation of its biocontrol action and inhibition calculated using an equation [14].

$$\begin{array}{c} \%\;inhibition=\left[D1-D2/D1\times 100\right] \end{array}$$

Where D1- diameter of radial growth of fungal pathogen alone, D2- diameter of radial growth of fungal pathogen in the presence of bacteria.

### Protocol validation

**Fig. 1:**
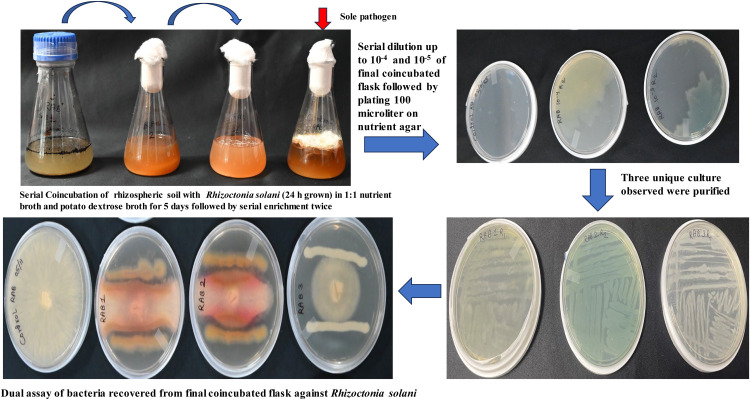
Isolation of antagonistic bacteria against *Rhizoctonia solani* through Serial Coincubation Enrichment Technique (SCET).

In the present study, we isolated three bacteria (RAB1, RAB2, and RAB3) from the *Rhizoctonia solani* pathogen grown in culture medium. Here, RAB 1 and RAB 2 showed antagonistic activity against the pathogen (Fig. 1). Fungal biomass was also significantly lower than that of *Rhizoctonia solani* grown alone, indicating that the bacteria present in the coincubated samples reduced fungal development (Table 2).

**Table 2:** Fungal dry weight of the sole pathogen compared with the pathogen coincubated.

Sample	*Rhizoctonia solani* (g)	*Sclerotium rolsii* (g)	*Colletotrichum truncatum* (g)
Sole pathogen	0.440 ± 0.019a	0.928 ± 0.065a	0.405 ± 0.008b
Coincubated	0.359 ± 0.018b	0.328 ± 0.039b	0.436 ± 0.016a
*p-* value	0.0067	<0.001	0.043

Where data shows (Mean ± Standard deviation, *n* = 3), followed by lowercase letters indicate statistically significant differences between treatments as analyzed using the Duncan Multiple Range Test (*p* < 0.05) through SPSS version 30.00.

**Fig. 2:**
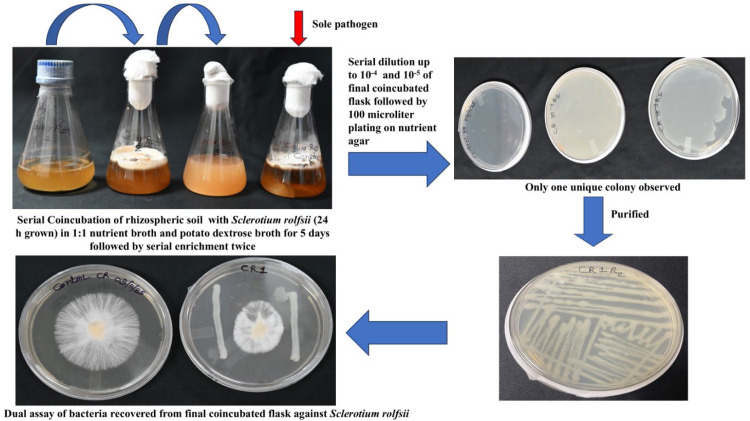
Isolation of antagonistic bacteria against *Sclerotium rolfsii* through Serial Coincubation Enrichment Technique (SCET).

**Fig. 3:**
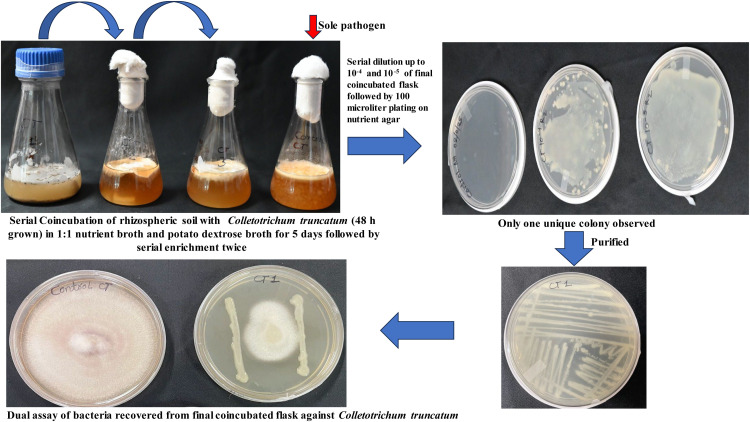
Isolation of antagonistic bacteria against *Colletotrichum truncatum* through Serial Coincubation Enrichment Technique (SCET).

Similarly, we obtained one bacterium each from fungal media containing *Sclerotium rolfsii* (Fig. 2) and *Colletotrichum tructatum* grown medium (Fig. 3), and named them CR1 and CT1, respectively. Among these, the bacteria isolated from *Colletotrichum truncatum* did not exhibit antagonism against the fungal phytopathogen, indicating that they were neutral. Further, the fungal biomass of the coincubated showed a non-significant reduction when compared to the sole growth of the fungus. At this stage, we can stop isolating and screening bacteria using the dual assay, as none exhibited antagonistic activity (Table 2).

**Fig. 4:**
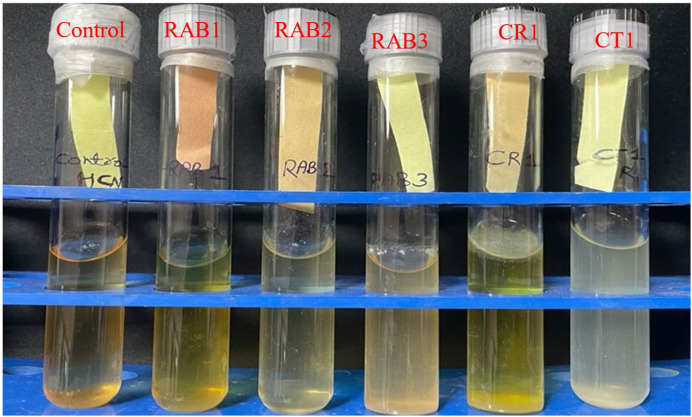
Qualitative hydrocyanic acid (HCN) production assay of recovered isolates. Here, yellow shows HCN negative and discolouration to brown shows HCN positive.

Using the described protocol, the recovered antagonistic bacteria inhibited the growth of *Rhizoctonia solani* by 50.83–57.91 %, *Sclerotium rolfsii* by 41 %, and *Colletotrichum truncatum* by 16.66 %. HCN production assay showed that two bacteria from *Rhizoctonia solani* (RAB1 and RAB2) and one from *Sclerotium rolfsii* (CR1) were HCN positive (Fig. 4). Here, RAB1 showed strong HCN production, whereas RAB2 and CR1 showed weak HCN production as indicated by the discolouration from yellow colour to brown. These results may be further validated by inoculating the recovered bacteria directly into potato dextrose broth containing the target pathogen and comparing the growth with that of the target pathogen grown alone in the medium.

In the present protocol, rhizospheric samples contain antagonistic bacteria against *Rhizoctonia solani* and *Sclerotium rolfsii*, resulting in a decrease in the fungal dry weight of the coincubated sample compared to that of the sole pathogen grown in the medium. In contrast, the coincubated sample of *Colletotrichum truncatum* has a greater fungal dry weight than the sole pathogen grown in the medium. Therefore, we can reject the sample (Table 2). This finding is also supported by the lowest percent inhibition by the recovered bacteria. Since we used technical replicates of the rhizospheric sample for all three pathogens in the present protocol, this indicates that the sample did not harbor antagonistic bacteria against *Colletotrichum truncatum*. All the bacteria recovered through SCET were tested for HCN production, indicating that only those bacteria that exhibited antagonistic activity against the target pathogen were HCN-positive.

The SCET may be used to isolate endophytic antagonistic bacteria from surface-sterilized plants by following the protocol. The SCET protocol can be used to isolate antagonists that act against multiple fungal phytopathogens when they are grown together as described. Furthermore, the SCET technique may be used to isolate antagonistic fungi, yeasts, or actinomycetes by designing media in a 1:1 ratio of the preferred medium for the antagonists to the target pathogen. Finally, the serially diluted coincubated sample was placed in media with antibiotics. eg. For the isolation of antagonistic actinomycetes from soil samples, the samples may be physically & chemically pretreated to promote the selective growth of actinomycetes [15]. Then, 1 g of pretreated soil sample may be coincubated directly with a 1:1 mixture of nutrient broth and actinomycetes broth that already contains a 24–48 h-grown fungal phytopathogen, and then enriched as described in the protocol. From the final coincubated flask, 1 ml of suspension may be serially diluted up to 10^−5^ and then 100 µl may be spread onto starch casein nitrate agar amended with fungistatin antibiotic, which might allow the growth of actinomycetes only [16].

Similarly, antagonistic fungi may be isolated by following the SCET protocol. Here, incubate 1 g of the soil sample in 100 ml of potato dextrose broth containing the broad-spectrum antibacterial antibiotic chloramphenicol at 50 µg ml^-1^, along with the already 24–48 h grown target fungal phytopathogen, and incubate for 5 days. Following this, 1 ml of the coincubated broth may be transferred to the media and incubated for 5 days; this step may be repeated twice for enrichment. Finally, the coincubated samples may be serially diluted to 10^−5^, and 100 µl of an aliquot is spread uniformly onto potato dextrose agar medium containing chloramphenicol, which kills both gram-positive and gram-negative bacteria and selectively allows the growth of the antagonistic fungi. On a petri dish, fungal antagonists would be dominant compared to the target fungal phytopathogen. Here, we can distinguish these two types of fungus based on fungal morphology, and this distinction can be further confirmed by microscopic examination of fungal hyphae and spores. In this case, comparing fungal biomass may not be an accurate way to screen samples for possible fungal antagonists.

### Limitation

In this protocol, some neutral bacteria also grow, which can thrive in the environments of the tested phytopathogen metabolites; therefore, they do not exhibit antagonistic properties. However, with the present protocol, only 1–2 bacteria appear per sample, which can be easily tested in a dual assay.

## CrediT authorship contribution statement

Hemant Singh Maheshwari, Laxman Singh Rajput, and Sanjeev Kumar: Conceptualization, methodology, and resources, writing original draft, analysis; Aakash Gour: conceptualization, investigation, and review of draft; Jeberlin Prabina Bright, Rakesh Kumar Singh, Kunwar Harendra Singh & Mahaveer Prasad Sharma: supervision and reviewing the final draft.

## Data availability

Data would be made available upon request to the corresponding author.

## Declaration of competing interest

The authors declare that they have no known competing financial interests or personal relationships that could have appeared to influence the work reported in this paper.
